# Pellagra; Its Symptoms and Treatment*Read before the Smith County Medical Society at its meeting in Tyler, September 8, 1914.

**Published:** 1915-04

**Authors:** L. P. Tenney

**Affiliations:** Troup, Texas


### Pellagra; Its Symptoms and Treatment.*

BY DR. L. P. TENNEY, TROUP, TEXAS.

No subject is of more importance to the medical profession today than that of pellagra; and this for several reasons: First it is a new disease so far as we, in this country, are concerned, and it is very important that we should familiarize ourselves with its symptomatology, in order that we may be able to make an early diagnosis, for on this point depends, in large measure, the success of treatment. Again it is important because of its rapid spread throughout the South especially.

The spread of pellagra is indeed assuming alarming proportions and demands of us as a profession that careful consideration that has ever characterized our efforts. Ten years ago this disease was hardly known in America; today hardly a physician even in the

*Read before the Smith County Medical Society at its meeting in Tyler, September 8, 1914.

remotest cross-roads district, but has under his care one or more such cases. Only a short time ago the Secretary of the Treasury of the United States recommended to Congress the appropriation of a sum of money sufficient to establish and maintain an institution for research work in regard to the possible cause and prevention of pellagra.

I shall dismiss the subject of etiology with a word, for as yet no one is able to say with certainty that this or that agency is the cause, nor to say that the disease is transmitted from the sick to the well by this or that method. The fact is, we must admit plainly we do not know the cause or mode of transmission, and until we do all efforts at prophylaxis are more or less futile. Suffice it to say my own opinion, based on a limited personal observation, is that we have to deal with a chemical poison, which gains entrance to the human economy through the alimentary tract. In support of this theory I mention that invariably the first and most prominent symptoms are those referred to the stomach and alimentary tract. Now whether this poison comes from spoiled corn, as is contended by the zeists, or from some other source, I do not know. There is a very important field for research work here, and he who discovers, beyond the peradventure of a doubt, this lurking demon, will have made his name immortal in the medical world.

I take it that we are all familiar with the train of symptoms that are so characteristic of pellagra; however, I feel it will not be amiss to call your attention to the more prominent symptoms.

The first symptom I call your attention to and the one which almost invariably precedes all others, is that referred to the alimentary tract; everyone of these patients will, under careful questioning, give a history of stomach troubles; indigestion, eructation of gas, a full feeling in the stomach after eating, more or less indefinite colicky pains and burning in the stomach or abdomen; an occasional attack of loose bowels, later becoming an annoying diarrhea, probably alternating with periods of constipation. Then comes a stomatitis; the mucous membranes of the soft palate, pharynx, gums and tongue are at least partially denuded, leaving the mouth and throat red and sore; at times so much so that one cannot eat solid food with any degree of comfort. The raw red tongue is very characteristic.

There is also, in the majority of cases, an increased activity of the salivary glands, resulting in a copious flow of saliva. I am of opinion, from observation, that the denudation of the epithelium extends throughout the entire alimentary tract to some extent.

There is an inability to digest and assimilate food, accompanied by gas formation and a burning sensation in the stomach and abdomen. In some severe cases I have seen the rectum and vagina raw and bleeding, and almost wholly devoid of its epithelial lining; one case in particular: a lady thought she had hemorrhoids, but upon examination we found the epithelial lining of the rectum almost destroyed, and as a consequence there was a partial prolapse of the rectum.

The next most important symptoms are those referable to the nervous system. The patient at first complains of an unexplained insomnia; lying awake at night for no apparent reason; then becoming irritable, excitable, very nervous.

These patients have said to me that they feel like they would "fly to pieces." These nervous manifestations grow gradually worse until the integrity of the central nerve cells is impaired and the mind is affected; these patients are very despondent and have all kinds of delusions, hallucinations, and oftentimes there is a suicidal tendency, and they require close watching.

One of the most characteristic nervous phenomena is the burning sensation; this may be referred to any part of the body, but is more common on the feet or hands; the particular location of this burning is not constant and may at one time be referred to the feet, at another time the hands may be complained of; or the patient may call your attention to a small spot no larger than a dollar, between the shoulders or on the chest or abdomen, that he describes as burning like a coal of fire.

As a result of the digestive and nervous disturbances we invariably find a progressive loss in weight, weakness, emaciation and vertigo. These patients have a peculiar staggering gait similar to that of a man under the influence of intoxicants.

The skin lesions I mention last because I consider them of least importance. The first appearance of the erythema presents itself in the form of a sunburn, usually on the backs of the hands, wrists and forearms; this is invariably bilateral, and may at first be so slight as not to cause any alarm; later in the course of the disease the skin assumes a dark brown, almost black hue; and this after a time scales off, leaving a raw surface; later on these raw surfaces heal, leaving a peculiar pinkish, shining scar. This erythema may appear on the face, forehead or back of the neck, and even here it is always bilateral.

One of the most striking peculiarities of pellagra is its seasonal recurrences; the exacerbations begin with the opening of the spring season, and all symptoms grow gradually worse during the heated season. If our patient survives through the summer all symptoms begin to abate with the arrival of cooler weather; during the winter months one feels much better, and the disease seems to lurk away, just as the snake hibernates, only to return the following spring with increased vigor; each recurrence being more virulent than the preceding attack.

The key to success in the treatment of these cases is early diagnosis and prompt and persistent application of the remedies at hand.

A few words as to diagnosis: There has been much discussion as to whether one can positively diagnose a case of pellagra in the absence of the skin lesions; and this has given rise to the question, is there such a thing as "pellagra sine pellagra" or pellagra without any skin manifestations? I believe that sooner or later every case will develop the characteristic erythema, but in many, if not the majority of instances, the skin lesions appear later in the course of the disease; and our patient may be in an almost hopeless condition before the skin symptoms appear. This point is very important when we take into consideration that early diagnosis is essential to success in the treatment.

I shall name the principal points in diagnosis, in what I consider the order of their importance.

First of all a history of stomach trouble, possibly dating back two or three or more years, and having a distinct seasonal recurrence; more pronounced in spring and summer, growing better and even disappearing entirely during the winter months; the peculiar griping accompanied by short periods of diarrhea, without any assignable cause, are very characteristic.

Next in importance are the nervous phenomena: sleeplessness, irritability, despondency, looking for and brooding over imaginary troubles: progressive loss in weight and strength.

The third and convincing point of evidence is the appearance of the peculiar bilateral erythema.

It is my opinion that we should go carefully into detail in our examination of all cases that give a history of chronic stomach or intestinal trouble, because we may be dealing with a case of pellagra in the early stages, or in the winter months when the more prominent symptoms are in abeyance.

As to treatment, many different remedies have been suggested and tried. The one remedy almost universally recommended by all authorities is arsenic. Its therapeutic value in this disease, so far, is not based upon any specific effect, but is more or less empirical. A number of remedies containing arsenic are now in use:

atoxyl, sodium caccodylate and soamin; all these contain from 22 to 26 per cent arsenic, and are put up in convenient form for hypodermic administration. Other remedies have been tried, but with one notable exception I think the preponderance of evidence is in favor of arsenic. This one exception has recently come to my attention through an article in our State *Journal*, reporting an address delivered by Dr. Isadore Dyer of New Orleans, at our last State meeting, in Houston. Dr. Dyer uses quinin hydrobromate, and claims for it excellent results.

Personally, in a limited experience, I have used soamin hypodermatically. This drug contains 22 per cent arsenic, and is put up in tablets of three and five grains each. My practice is to begin with one three-grain tablet hypodermatically every other day, and after a week or ten days, every day for a week longer, then every other day for a short time, when I lengthen the intervals to, say, twice a week, continuing this indefinitely or until symptoms clear up; then I continue its use once a week for some two or three months longer. The idea is to keep the patient saturated with arsenic, and it is surprising how much they can stand.

In addition to the hypodermic medication, after patient's mouth and digestive tract begin to show marked improvement, which is generally within two weeks, I then give Fowler's solution internally, commencing with five drops three times daily, and increasing one drop each day up to twelve or even fifteen drops three times daily; then returning to the original dose of five drops and again increasing by one drop daily to the maximum limit. Some patients, of course, will not stand as much as others, and in this we are guided by the well known and easily recognized symptoms of arsenic saturation. With some it may be necessary at times to discontinue altogether the use of Fowler's solution for several days or even a week.

Later after patient is improving I give tonics; of these I consider cod liver oil most valuable; also iron in some form should be given.

Before leaving the subject of drug medication I want to say that I always try to impress my patients with the idea that they must not consider themselves entirely well until they have passed through two summers without a return of the symptoms. And in order to be on the safe side, I direct my patients to return for further treatment early in the spring of the following year, before the hot weather has set in; at which time I repeat the hypodermic administration of soamin every other day for about one month, and then give Fowler's solution as outlined above for another month.

We also insist on a liberal diet, for it is imperative that these patients receive sufficient nourishment to replace the lost strength and flesh. To this end I insist on patient eating plenty of good wholesome food, especially milk and eggs. I do not restrict my patients to any particular diet list, but give them a free rein, only avoiding a few of the more indigestible articles, such as fried meats, etc.

I also urge these patients to eat from one to two or three lemons daily, as I find that this to some extent supplies the deficiency in acids in the gastric juices so common in these cases.

For the distressing stomach symptoms, especially the tendency to gas formation, I find hydrochloric acid in ten-drop doses immediately after eating very beneficial.

From my experience, covering a number of cases, within the past two years, I am convinced that with a careful, watchful and persistent application of the remedies as herein outlined, the prognosis is, in the large majority of cases, favorable.

---

COVERING UP MISTAKES.—A pompous physician who was inclined to criticise others, was watching a stonemason build a fence for his neighbor. He thought the mason used too much mortar.

"Jim," he said, "mortar covers up a good many mistakes, doesn't it?"

"Yes, doctor," calmly replied the mason, "and so does the spade."—*Ex.*

---

TRUTH WORTH $50 TO HIM.—Dr. George Owens was charged in Flatbush court with speeding his auto thirty miles an hour along Ocean boulevard.

"I admit the charge," said the physician. "It was very cold and I was anxious to get home."

"You are the first doctor who ever told the truth in a case of this kind," said Magistrate Reynolds. "Instead of the $50 fine agreed upon by the board of magistrates for all self-confessed speeders, I am going to let you off with a $25 fine."

Dr. Owens took a roll of bills from his pocket and was about to pay the amount when the magistrate again spoke.

"On second thought I am going to let you go with a suspended sentence," he said. "Such truthfulness is to be encouraged."—*New York Sun.*

